# CO_2_ Binding at Gold Cluster Cations: An Infrared Free‐Electron Laser Spectroscopic Investigation

**DOI:** 10.1002/cphc.70623

**Published:** 2026-11-10

**Authors:** Christian T. Haakansson, Peter T. Rubli, David J. Vesty, Olga V. Lushchikova, Joost M. Bakker, Stuart R. Mackenzie

**Affiliations:** ^1^ Department of Chemistry Chemistry Research Laboratory University of Oxford Oxford UK; ^2^ HFML‐FELIX Nijmegen The Netherlands; ^3^ Institute for Molecules and Materials Radboud University Nijmegen The Netherlands

**Keywords:** broadband absorption, CO_2_ activation and sequestration, gold nanoparticles, infrared spectroscopy, metal cluster

## Abstract

Understanding the fundamental binding motifs of carbon dioxide is key to its efficient sequestration and/or molecular activation. In this context, gas‐phase metal clusters represent key model systems for the design of next generation materials. Infrared spectroscopy, especially using free‐electron lasers, provides a direct probe of molecular structure across a range of wavelengths inaccessible by tabletop light sources. Here, we report the investigation of CO_2_ binding to isolated gold cluster cations, Au_*n*_^+^ (*n* = 4–11), using the FELICE light source, with structural assignments aided by quantum chemical calculations. In order to capture the well‐known Fermi dyad arising from resonance between the symmetric stretch and the overtone of the bend, an anharmonic vibrational treatment has been employed. Binding to a gold cluster cation only weakly perturbs the CO_2_ which retains its linear geometry. Coordination occurs exclusively via an oxygen atom and a single gold atom of the cluster (η^1^‐O), with very weak molecular activation observed. Successive CO_2_ molecules bind in similar fashion to the first leading to multiply‐solvated clusters.

## Graphical abstract

Structures and binding motifs of carbon dioxide adsorption to gas‐phase gold cluster cations have been determined by infrared free electron laser spectroscopy supported by quantum chemical calculations.

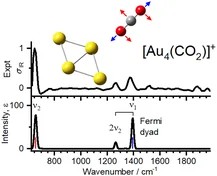

## 1 Introduction

The link between increasing atmospheric carbon dioxide (CO_2_) concentration and global warming, as well as associated climate change, has presented a global challenge to develop our understanding of chemical activation and sequestration of CO_2_ [1, 2]. A key first step towards utilization of CO_2_ as a cheap feedstock is the binding or fixation of the molecule to some sequestering material. Any detailed understanding of the mechanisms and motifs involved in CO_2_ adsorption requires structure‐sensitive gas‐phase studies free from the complexity introduced by solvents and support material [3]. Atomically precise clusters offer the opportunity to explore the cluster size dependance of important benchmark properties such as molecular binding energies and/or chemical activation. For example, they provide a potential route to determine the cluster size regime in which the CO_2_ binding energy lies in the 0.25–0.5 eV range (intermediate between physisorption and chemisorption), considered ideal for practical carbon capture and storage. In the small size regime (<30 atoms), metal clusters also provide a rigorous and tractable test for modern quantum chemical calculations of structures and properties.

The simplest metal‐CO_2_ binding motifs of all are found in gas‐phase M^±^(CO_2_)_*m*_ ion‐molecule complexes whose structures have been determined by infrared spectroscopy. M^+^–CO_2_ interactions are characterized by weak, linear bonding with minimal molecular perturbation [4, 5, 6, 7, 8, 9], and such species are appealing as model systems for CO_2_ fixation. Simple metal oxide cations, MO^+^, especially lanthanide and actinide oxides, can drive activation via formation of radical carbonate‐like moieties [10, 11, 12, 13]. Metal anion complexes, M^–^–CO_2_, exhibit extensive molecular activation resulting from efficient electron donation into CO_2_ antibonding 2*p*π* orbitals, which leads to a rich array of structural binding motifs, especially with multiple CO_2_ ligands [14, 15, 16, 17, 18, 19].

Gas‐phase metal clusters represent a unique class of species whose interactions with small molecules can show important differences from those of both simplified single‐metal ions and extended surfaces [20, 21, 22]. Small clusters can also exhibit unique size‐dependent reactivity not present in the bulk [23, 24]. Isolated metal clusters also have the advantage of being amenable to ultrasensitive mass spectrometric detection, which allows their structures to be studied by infrared multiple‐photon dissociation (IRMPD) spectroscopy [25]. However, despite the need for a clearer understanding of CO_2_ fixation, only limited studies of the binding motifs in metal cluster‐CO_2_ species have been reported [26, 27, 28, 29, 30, 31, 32, 33, 34, 35, 36]. In terms of homogeneous metal clusters, CO_2_ binding has been investigated on Co_*n*_^–^ [27], Pt_*n*_^–^ [28], Rh_*n*_^*–*^ [33], Co_*n*_^+^ [35], and Cu_*n*_^+^ [36]. Binding to the anionic clusters can lead to effective (strongly size‐dependent) CO_2_ activation, resulting in significant spectral shifts in the CO_2_ vibrational bands (notably a blueshifted bend and redshifted antisymmetric stretch) [28, 33]. In the extreme limit, dissociative adsorption produces a characteristic oxo‐metal carbonyl (OM_*n*_^±^–CO) absorption band [27, 28, 33]. By contrast, CO_2_ binding to metal cluster cations typically results in chemically inert, linear binding, signified by the observation of a Fermi dyad that arises from a Fermi resonance between the CO_2_ symmetric stretch and first overtone of the bend [32, 36, 37].

Unlike the famously inert bulk gold, atomically precise gold clusters can exhibit marked, and often selective, catalytic activity [24, 38, 39, 40], which is believed to originate from relativistic effects that drive the 5*d* orbitals closer in energy to the 6*s* [41, 42]. Nanoparticulate gold has been shown to be catalytically active in chemical reactions such as CO oxidation and the water–gas shift reaction [43, 44]. Moreover, electrochemical reduction of CO_2_ occurs with a Faradaic efficiency of 91% at gold electrodes [45], with oxide‐derived nanoparticles further reducing the required overpotential [46]. Metal organic frameworks (MOFs) have potential for use in reversible carbon capture and sequestration provided appropriate binding energies are determined [47], and gold(I)‐based MOFs have already been developed as catalysts for alkyne carboxylation using CO_2_ [48].

The structures of naked gold clusters in the gas‐phase have been studied using a wide range of experimental techniques [49, 50, 51, 52, 53, 54]. Ion mobility mass spectrometry quickly revealed that the smallest clusters exhibit unusual planar structures arising from efficient *s–d* hybridization. For the cluster cations, Au_*n*_^+^, the switch from planar to three‐dimensional structures occurs around *n* = 8 [55], a result later confirmed by IRMPD spectroscopy [56].

Molecular activation at gold cluster cations has been studied by infrared dissociation spectroscopy [25] for a range of small molecules, including CO [57], NO [58], O_2_ [59], N_2_O [60, 61], OCS [61, 62], and C_2_H_2_ [63]. These molecules typically bind linearly to a single gold atom of the cluster, with the exception of acetylene, for which binding occurs orthogonal to the C─C bond [63]. Several of these studies reveal strong odd‐even cluster size alternations reflecting the closed‐shell/open‐shell nature of the underlying Au_*n*_^+^ cluster. This is particularly clear in NO binding at gold cluster cations where the NO stretch vibrational band is typically redshifted by 60–80 cm^−1^ on Au_*n*_^+^ (*n* = 6, 8, 10, 12…) clusters but very close to the free NO stretch frequency on odd *n* clusters Au_*n*_^+^ (*n* = 5, 7, 9, 11, 13…) [58]. More subtle electronic effects are observed in the resonant IR fragmentation dynamics of [Au_*n*_OCS]^+^ clusters: only simple IR‐induced OCS loss occurs from Au_*n*_OCS^+^ (*n* odd), whilst unusual IR–driven bond‐breaking chemistry (leading to Au_*n*_S^+^ production and CO loss) is observed for Au_*n*_OCS^+^ (*n* even) [62].

Here, we report a combined experimental and computational investigation into the nature of CO_2_ binding to cationic gold clusters, Au_*n*_^+^ (*n* = 4–11). IRMPD spectra have been recorded using the Free‐Electron Laser for IntraCavity Experiments (FELICE) at HFML–FELIX in Nijmegen, the Netherlands. To aid in the assignment of the vibrational spectra, simulated spectra of energetically low‐lying structural isomers have been calculated using generalized second‐order vibrational perturbation theory, in order to capture the well‐known Fermi dyad.

## 2 Experimental and Computational Methods

All experiments were performed utilizing the molecular beam apparatus coupled to the FELICE beamline at the HFML–FELIX facility in Nijmegen, the Netherlands. The instrument, including its cluster source, as well as the free‐electron laser (FEL) beamline have been described in detail previously [35, 64, 65, 66]. One aspect of the FEL which will prove pertinent later is that weak harmonics of the fundamental frequency are present in the beam [67]. Briefly, gold clusters were produced by pulsed laser ablation (Nd:YAG laser, 532 nm, ~20 mJ, 8 ns, 10 Hz) of a translating stage containing a gold target in the presence of a helium carrier gas expansion from a pulsed valve (Jordan TOF Products, Inc., stagnation pressure of 8 bar).

CO_2_ gas was introduced into the clustering channel downstream of the ablation via a secondary valve. The resultant cationic cluster distribution was detected mass spectrometrically in an orthogonal reflectron time‐of‐flight mass spectrometer. The cluster source operates at 10 Hz, with IRMPD spectra produced by exposing alternate cluster pulses to the 5 Hz FELICE beam as it scans in the spectral range of 630–1980 cm^−1^. The spectra are recorded as depletions in the parent cluster ion signals and are presented as normalized absorption cross‐sections, *σ*_IR_, generated at a particular FEL wavelength:

(1) $$\sigma_{\mathrm{IR}}=-\frac{1}{\phi \left(\lambda \right)}\ln\left(\frac{I_{\mathrm{On}}}{I_{\mathrm{Off}}}\right),$$

where *I*_On_ and *I*_Off_ are FEL‐on and FEL‐off ion signals respectively, and ϕ(λ) is the wavelength‐dependent, intracavity, FELICE macropulse energy. To determine the intracavity power for this normalization, a small fraction of the light from the FELICE cavity is directed onto a power meter. Similarly, wavelength calibration was performed by directing the outcoupled light onto a grating spectrometer.

In aid of structurally assigning the experimental spectra, simulated IR spectra of energetically low‐lying isomers have been determined with density functional theory (DFT) using the Gaussian 16 software package [68]. A particular focus was placed on determining the CO_2_ binding motif. The UB3P86 [69, 70]/def2‐TZVPPD [71, 72, 73] level of theory was used, a choice guided by its performance in previous metal cluster–adsorbate studies [33, 74]. In addition to chemical intuition, a modified Kick3 algorithm was employed in order to generate starting structures and thus allow for a broader search of conformer space [75, 76]. In order to facilitate comparison between experiment and theory and reproduce the CO_2_ Fermi dyad, the simulated IR spectral frequencies have been treated anharmonically by way of a generalized second‐order vibrational perturbation theory (GVPT2) [77], as implemented in Gaussian 16. In addition, the binding energies of CO_2_ to various gold cluster cations have been determined (i.e., binding energy = –*E*_ads_ = –[*E*_0_([Au_*n*_(CO_2_))]^+^ – (*E*_0_(CO_2_) + *E*_0_(Au_*n*_^+^))]). All energies include vibrational zero‐point corrections.

## 3 Results and Discussion

A representative mass spectrum of the cluster distribution generated in the source is shown in Figure 1, with assignments based on molecular formulas and the square bracket notation used to reflect no a priori knowledge of the chemical structure. Bare gold cluster cations, Au_*n*_^+^, and [Au_*n*_(CO_2_)]^+^ (*n* = 4–11) clusters dominate the mass spectrum from *n* > 4 and are labeled. Evidence of clusters binding multiple CO_2_ molecules, [Au_*n*_(CO_2_)_*m*_]^+^ is also observed. Importantly, there is no trace of Au_*n*_O^+^ peaks, which could signify O–atom transfer reactions between gold clusters and CO_2_ molecules followed by CO elimination. Contaminant peaks including [Au_*n*_(H_2_O)]^+^ and mixed‐ligand species, involving both CO_2_ and H_2_O, [Au_*n*_(CO_2_)_*x*_(H_2_O)_*y*_]^+^, are observed in the mass spectrum, as indicated with asterisks in Figure 1. The mixed‐ligand species will be the focus of a future publication. We have attempted to estimate relative CO_2_ sticking coefficients (i.e., relative rate constants associated with the Au_*n*_^+^ + CO_2_ → [Au_*n*_(CO_2_)]^+^ reaction) in Figure S1. This shows a smooth rapid decrease with increasing cluster size, likely reflecting a dilution of the positive charge as *n* increases.

**FIGURE 1:**
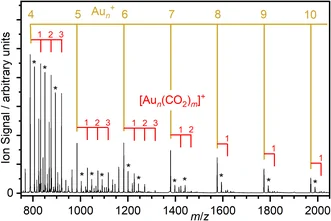
Time‐of‐flight mass spectrum produced by ablation of a gold target in the presence of He carrier gas, with CO_2_ introduced through a secondary nozzle. The naked Au_*n*_^+^ and [Au_*n*_(CO_2_)_*m*_]^+^ clusters of interest in this work are indicated in gold (*n*) and red (*m*), respectively, where the gold numbers denote the number of Au atoms (*n*) and the red numbers denote the number of attached CO_2_ molecules (*m*). Asterisks indicate water‐,containing species.

### 3.1 Spectra and Structural Assignment of [Au_*n*_(CO_2_)]^+^ (*n* = 4–11)

Figure 2 shows the IRMPD spectra of the [Au_*n*_(CO_2_)]^+^ (*n* = 4–11) cluster series in the spectral region 630–1980 cm^−1^ recorded in depletion of the parent ion signal, power‐corrected according to Equation (1), and normalized to the spectral peak at ~650 cm^−1^, which is common to all spectra. The peaks observed correspond to resonant infrared absorption, which leads to loss of CO_2_ from the precursor cluster. Several features, including bands at 650, 1270, and 1390 cm^−1^, are present in all spectra. The uniquely different [Au_10_(CO_2_)]^+^ spectrum is discussed below, but otherwise the general spectral similarity for different cluster sizes immediately suggests a common CO_2_ binding motif on all clusters studied. By design, this study, focusing on molecular vibrations in the mid‐infrared, yields more information on the CO_2_ binding motif than it does on the structure of the underlying gold clusters. For the latter, a far‐infrared study involving inert messenger tags would likely be required.

**FIGURE 2:**
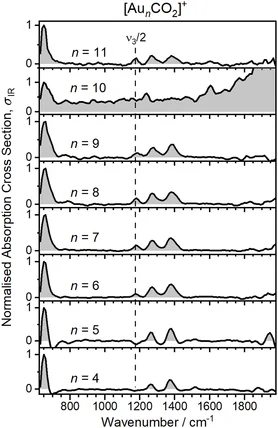
Normalized infrared action spectra of [Au_*n*_(CO_2_)]^+^ (*n* = 4–11) clusters recorded in depletion of the parent ion species. Gray shading indicates infrared absorption and helps highlight the broad absorption observed across the full wavelength range studied for [Au_10_CO_2_]^+^. The dashed line marks one half of the CO_2_ ν_3_ fundamental frequency (1175 cm^−1^), observed as a result of the second harmonic in the FEL beam.

The spectral bands observed are readily assigned based on previous M_*n*_(CO_2_)^+^ studies, especially CO_2_ binding at cobalt and copper cluster cations [35, 36] and at metal oxide cluster cations [31, 32]. The 650 cm^−1^ band is assigned to the CO_2_ bending vibration, observed at 667 cm^−1^ in the free molecule. The persistent pair of bands at 1270 and 1390 cm^−1^ is the well‐known Fermi dyad, which arises as a result of the accidental degeneracy of the symmetric stretch ν_1_ (IR active upon binding to the cluster) and first overtone of the bending mode ν_2_. The remaining band observed in almost all spectra at 1175 cm^−1^ is observed at exactly half the wavenumber of the strongly IR‐active antisymmetric stretching mode (ν_3_) and arises due to the presence of the overtone in the FEL cavity [67]. This feature was previously observed in FELICE studies of CO_2_ binding to Cu_*n*_^+^ and Co_*n*_^+^ clusters [35, 36]. With this understanding, it is labeled as (ν_3_/2) and provides information on the ν_3_ mode (2349 cm^−1^ in free CO_2_) even though the fundamental lies outside of this spectral region. One apparent trend is observed in which the relative intensity and linewidth of the Fermi resonance peaks increase slightly with respect to other spectral peaks with increasing cluster size. This is partly due to the spectra having been normalized to the peak intensity of the bend fundamental, whose linewidth also changes. It is, however, also the case that larger clusters provide more potential CO_2_ binding sites, leading to slight shifts in vibrational frequencies [72].

The spectrum of one cluster is markedly different from all others. The [Au_10_(CO_2_)]^+^ spectrum exhibits a broad, largely featureless background depletion across the whole range, which becomes ever stronger toward the blue end of the region scanned. This feature has been observed previously in the spectrum of Au_10_^+^ with various closed‐shell adsorbates including Au_10_(OCS)^+^ [62], Au_10_(N_2_O)^+^ and Au_10_Ar^+^ [61]. We have previously interpreted this band as evidence for a very low‐lying electronic transition in Au_10_^+^ arising from its unusual distorted tetrahedral structure [61]. We have shown previously that pumping this electronic transition can drive bond breaking in the molecular adsorbate [61], but in the present case, the only fragmentation channel observed is simple CO_2_ loss (i.e., ligand loss).

In order to verify the assignment of the spectral bands and hence confirm the associated CO_2_ binding motif, the experimental IRMPD spectra are compared with anharmonically simulated IR spectra of low‐energy structures calculated at the UB3P86/def2‐TZVPPD level of theory. Such a comparison for the representative [Au_4_(CO_2_)]^+^, [Au_5_(CO_2_)]^+^, and [Au_6_(CO_2_)]^+^ clusters is shown in Figure 3. In Figure 3b,c ν_3_/2 (ν_3_ = 2349 cm^−1^ for free CO_2_) is also indicated for completeness. This band is barely visible in the [Au_4_(CO_2_)]^+^ spectrum and appears as a dip in the depletion spectrum of [Au_5_(CO_2_)]^+^ reflecting enhancement in this channel due to CO_2_ loss from [Au_5_(CO_2_)_2_]^+^ (see Figure 4). Similar effects are observed at around 700 cm^−1^. In each case, the bare gold cluster structures agree qualitatively with previous experimental structural determinations [55, 56]. The advantage of the anharmonic simulation is clear in that it reproduces the CO_2_ Fermi dyad, which is absent in harmonic calculations.

**FIGURE 3:**
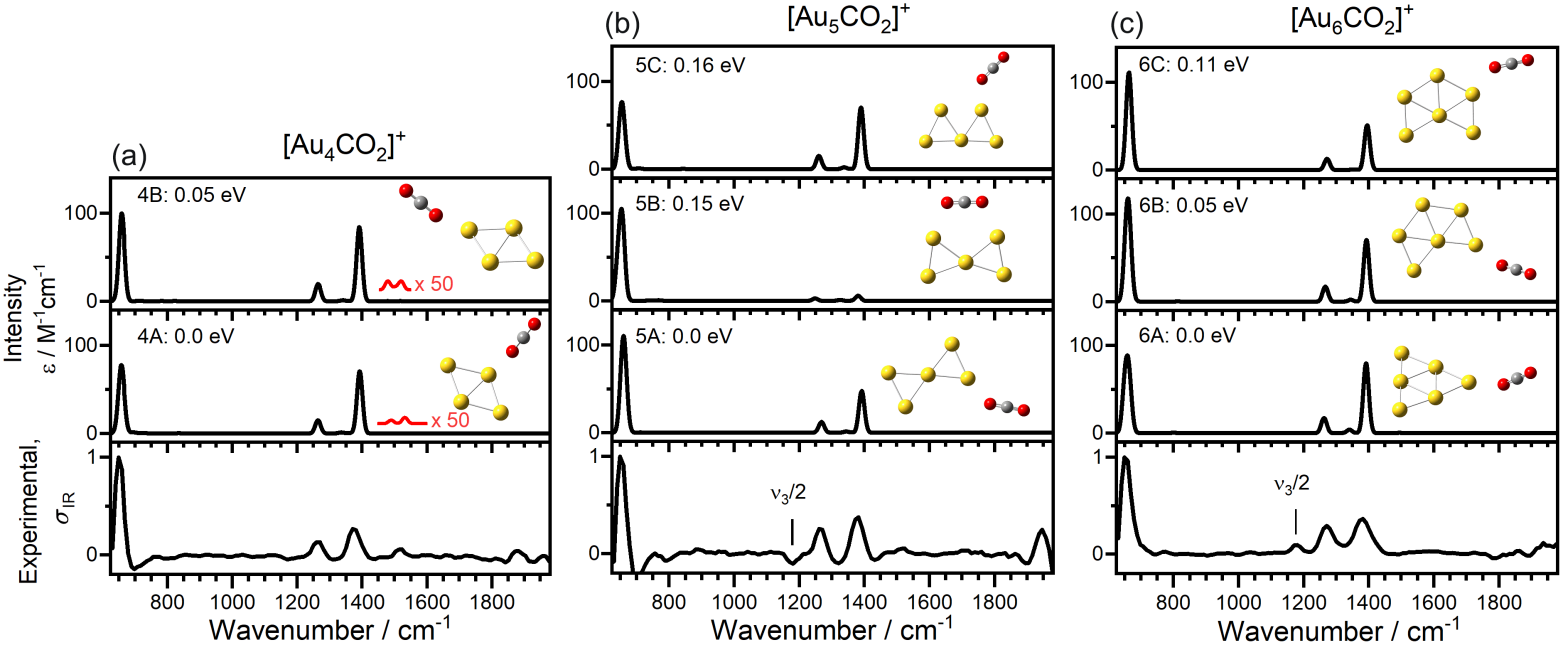
Comparison of experimental IRMPD spectra and simulated anharmonic spectra for low‐energy structures calculated at the UB3P86/def2‐TZVPPD level of theory for [Au_*n*_(CO_2_)]^+^ (*n* = 4 – 6). In each case, the relative energy of different isomers has been provided. For *n* = 4, 6 (a) and (c) all calculated structures are doublet spin states, and for *n* = 5 (b) structures, singlets. Calculated bands have been convoluted with a Gaussian line shape of 20 cm^−1^ FWHM, and in (b), (c) the observed position of ν_3_/2 is marked (ν_3_ = 2349 cm^−1^ in free CO_2_, see text for details).

In the case of [Au_4_(CO_2_)]^+^ (Figure 3a), CO_2_ can bind to either of the two unique sites of the rhombus‐shaped gold tetramer in a manner as previously observed in N_2_O binding to Au_4_^+^ [60]. These two isomers are near isoenergetic, and spectrally, the two motifs are too similar to distinguish experimentally, with both conformers showing excellent agreement with the experimental spectrum. Evidence of an additional weak band can be found in the experimental spectrum at 1500 cm^−1^. The simulated spectra reproduce this band (see Figure 3a), which corresponds to the CO_2_ symmetric stretch in combination with a low‐energy cluster breathing mode, but significantly underestimate its oscillator strength. The small feature observed at ~1900 cm^−1^ is likely the second overtone of the CO_2_ bending vibration.

The global minimum structure of the Au_5_^+^ clusters is a bow tie arrangement [56, 62]. This offers multiple, near equivalent potential binding sites for CO_2_ and as a result, no single assignment can be made for [Au_5_(CO_2_)]^+^ (Figure 3b). Good agreement with the experimental spectrum is nonetheless observed for several low‐energy isomers, all of which have CO_2_ binding in η^1^‐O motif (i.e., via the O atom to a single Au atom). In addition to the Fermi dyad, the experimental spectrum shows a weak band at ~1945 cm^−1^, which could be the second overtone of the CO_2_ bending vibration or a bend‐stretch combination band. Dips in the [Au_5_(CO_2_)]^+^ spectrum at 700 and 1175 cm^−1^ arise as a result of CO_2_ loss from [Au_5_(CO_2_)_2_]^+^ or [Au_5_(CO_2_)(H_2_O)]^+^.

For [Au_6_(CO_2_)]^+^ (Figure 3c), once more several structural isomers provide good matches for the experimental spectrum, all of which have CO_2_ bound linearly (η^1^‐O). At least two low‐lying structures for Au_6_^+^ are identified, but again, the CO_2_ spectral bands provide poor diagnostics of the underlying cluster structure. The *n* = 6 spectrum is the first in the series to show clearly the (ν_3_/2) band arising from the FEL harmonic. This feature, at ~1175 cm^−1^ is observed in all larger cluster structures except *n* = 10. Overall, the simulated anharmonic IR spectra reproduce the experimental spectra well, confirming the assignment of inert and linear (η^1^‐O) CO_2_ binding.

### 3.2 Binding Additional CO_2_

Upon increasing the partial pressure of CO_2_ in the cluster source, sufficient clusters of the form [Au_*n*_(CO_2_)_2,3_]^+^ were generated to allow collection of their respective IRMPD spectra, shown in Figure 4. In keeping with their single CO_2_ counterparts, the spectra are remarkably consistent irrespective of cluster size or number of CO_2_ ligands. In fact, the bands appear in essentially the same positions (approximately 650, 1180, 1270, and 1390 cm^−1^) as those shown earlier in Figure 2. This, in turn, suggests that successive CO_2_ molecules are bound to the cluster in identical (η^1^‐O) binding motifs to the first, that is, linearly to atom‐binding sites. In other words, the band assignments can be made from lowest to highest energy as the CO_2_ bend, the antisymmetric stretch via ν_3_/2, and the Fermi dyad of the bend overtone and symmetric stretch. As more CO_2_ molecules bind, the spectral bands start to show evidence of substructure (noticeable in the [Au_6_(CO_2_)_3_]^+^ spectrum, perhaps reflecting multiple isomers and/or CO_2_ binding sites.

**FIGURE 4:**
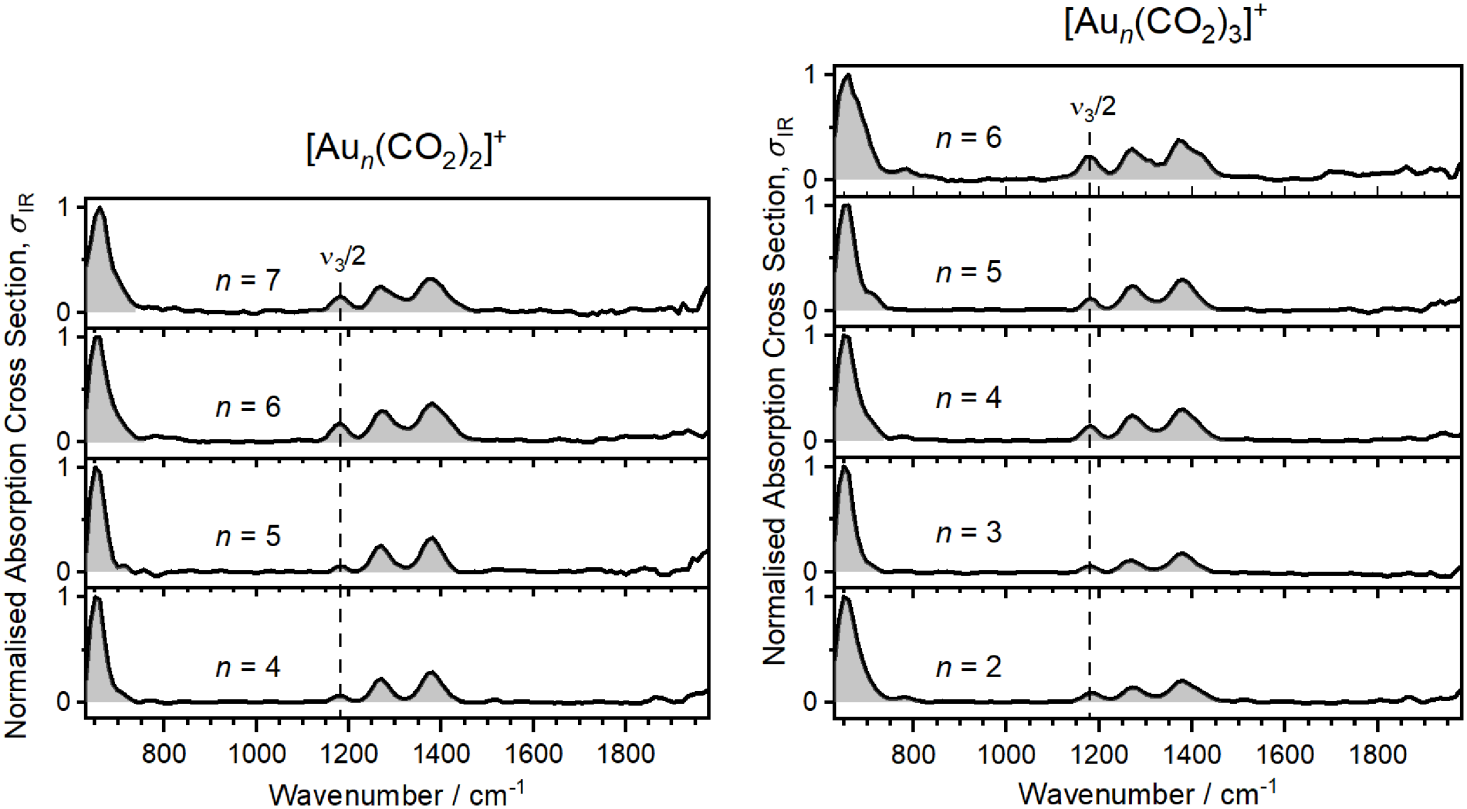
Normalized infrared action spectra of [Au_*n*_(CO_2_)_2_]^+^ (*n* = 4–7) and [Au_*n*_(CO_2_)_3_]^+^ (*n* = 2–6) clusters recorded in depletion of the parent ion species. Gray shading indicates infrared absorption.

As with the single CO_2_ clusters, structural assignment has been attempted through comparison with simulated anharmonic IR spectra of low‐energy calculated structures. Due to the striking similarity across all spectra recorded, Figure 5 shows the comparison for the prototypical Au_4_^+^. For both [Au_4_(CO_2_)_2_]^+^ and [Au_4_(CO_2_)_3_]^+^ (Figure 5a,b), each additional CO_2_ molecule is observed to bind to a new gold atom in an inert linear manner through the oxygen atom. The putative assignments made previously are confirmed, with the anharmonic calculations once again reproducing the experimental spectrum very well and indicating the presence of the now‐familiar CO_2_ bend and the Fermi dyad.

**FIGURE 5:**
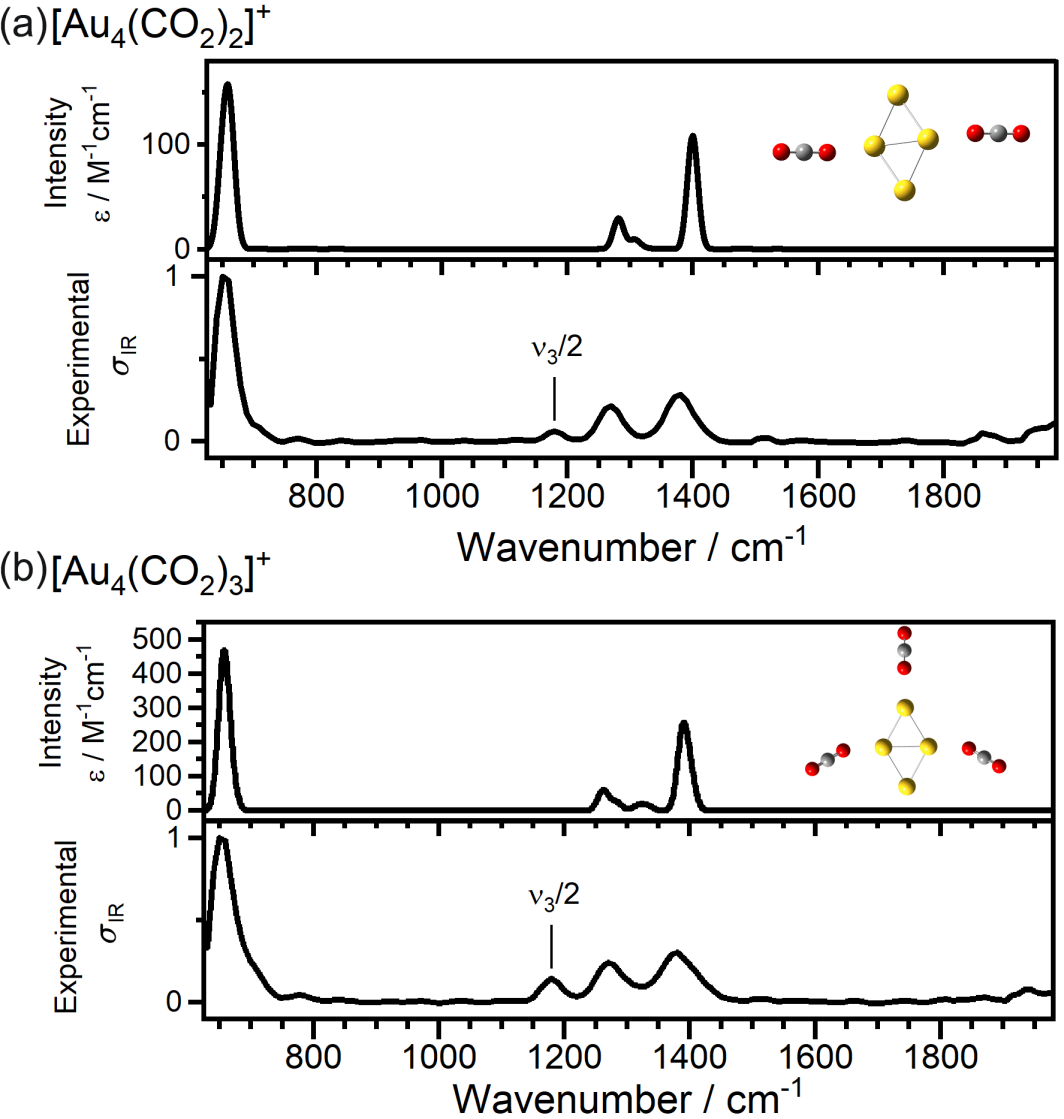
Comparison of experimental IRMPD spectra and simulated IR spectra for (a) [Au_4_(CO_2_)_2_]^+^ and (b) [Au_4_(CO_2_)_3_]^+^. In each case, only the calculated global minimum is shown. All calculated structures are doublet spin‐states. Calculated bands have been convoluted with a Gaussian line shape of 20 cm^−1^ FWHM.

### 3.3 CO_2_ Binding Energy to Gold Clusters

The initial binding of carbon dioxide to a material is a required first step before potential reactivity of the molecule. In addition to yielding binding motifs for CO_2_ fixation, the DFT simulations yield information on the strength of CO_2_ binding to the gold clusters (Table 1). The strongest binding (0.54 eV) is to the smallest cluster calculated, Au_3_^+^, with the binding energy dropping as the cluster size increases until Au_10_^+^ (0.24 eV). This effect likely originates in the evolution of electronic structure as the unit positive charge is diluted across more atoms as the clusters grow in size, thereby reducing the electron donation from the CO_2_ which dominates the binding. There is some evidence for marginally weaker CO_2_ binding to open‐shell Au_*n*_^+^ clusters (*n* even) than to adjacent closed‐shell (*n* odd) clusters. On the Au_4_^+^ cluster, perhaps unsurprisingly, the first CO_2_ molecule is calculated to bind slightly more strongly than subsequent molecules, each of which binds approximately 50 meV more weakly than the last. Interestingly, these binding energies lie within the range considered ideal for CO_2_ sequestration in MOFs and, although calculated for isolated clusters, suggest cluster size could in principle be used to tune the efficiency/reversibility of CO_2_ uptake in, for example, a cluster‐decorated MOF [47].

**TABLE 1:** Calculated CO_2_ binding energies with respect to each cluster size studied. The binding energy is observed to decrease both with increasing cluster size and with increasing ligation of a specific cluster size.

[Au_*n*_(CO_2_)_*m*_]^+^	CO_2_ binding energy, eV		
	*m* = 1	*m* = 2	*m* = 3
*n* = 3	0.54		
*n* = 4	0.40	0.36	0.29
*n* = 5	0.41		
*n* = 6	0.36		
*n* = 10	0.24		

## 4 Summary and Conclusions

In summary, free‐electron laser‐based IRMPD spectroscopy has been applied to determine the nature of CO_2_ binding to gold cluster cations. On all clusters, CO_2_ binds linearly in an inert (η^1^‐O) motif, with little evidence of activation. The observed vibrational bands are readily assigned as the CO_2_ bend, the CO_2_ antisymmetric stretch (observed at ν_3_/2 as a result of the presence of harmonics in the FEL beam), and a Fermi dyad arising from interaction between the overtone of the bend and the infrared‐active symmetric stretch. The structural and binding motif assignments are confirmed through DFT simulations and associated simulated IR spectra that account for anharmonicity. The latter show excellent overall agreement with the experimental spectra including reproduction of the Fermi resonance. The spectrum of the [Au_10_(CO_2_)]^+^ cluster is anomalous to all others in that it exhibits a broad infrared absorption across the whole spectral region studied, which has been documented previously [61].

Many of the gold cluster cations bind multiple CO_2_ molecules and the spectra of these species have also been recorded. Successive carbon dioxide molecules adsorb linearly to single gold‐atom atop sites, leading to remarkably similar spectra for all cluster sizes and CO_2_ coverages. Calculated CO_2_ binding energies lie within the 0.5–0.25 eV range, considered ideal for carbon capture/sequestration. These range from 0.54 eV for CO_2_ binding to Au_3_^+^ to 0.24 eV for Au_10_^+^, with subsequent molecules binding slightly less strongly.

## Funding

This work was supported by the EPSRC under program grant EP/T021675.

## Conflicts of Interest

The authors declare no conflicts of interest.

## Supporting information

Supporting information includes relative sticking coefficients of successive cluster size with respect to CO_2_, geometric structures for assigned isomers, as well as vibrational data associated with assigned isomers. The experimental data presented here are publicly available from the Oxford Research Archive (DOI: https://doi.org/10.5587/ora‐go6nvkyvj (to follow)).

## Data Availability

The data that support the findings of this study are openly available in Oxford Research Archive at ora.ox.ac.uk. (DOI: https://doi.org/10.5587/ora‐go6nvkyvj To be added upon publication).
