# Supplementary material for: CO2 Binding at Gold Cluster Cations: An Infrared Free‐Electron Laser Spectroscopic Investigation
Source: Chemphyschem. 2026 Nov 10;27(21):e70623. doi: 10.1002/cphc.70623 (PMC13752460; doi:10.1002/cphc.70623)
Supplement: Supplementary file 1 [file CPHC-27-e70623-s001.pdf]

CO_2 Binding at Gold Cluster Cations: An Infrared Free-Electron Laser Spectroscopic Investigation

*Christian T. Haakansson,[1] Peter T. Rubli,[1] David J. Vesty,[1] Olga V. Lushchikova,[2] Joost M. Bakker,[2,3] and Stuart R. Mackenzie[1]**

[1] Department of Chemistry, University of Oxford, Chemistry Research Laboratory, Mansfield Road, Oxford OX1 3TA, United Kingdom

[2] HFML-FELIX, Toernooiveld 7, Nijmegen 6525 ED, The Netherlands

[3] Institute for Molecules and Materials, Radboud University, Heyendaalseweg 135, Nijmegen 6525 AJ, The Netherlands

Supporting Information

The supporting information presented here comprises of additional reactivity data associated with the reactivity of CO_2 and Au_n^+ as well as results of DFT calculations performed on $[Au_n(CO_2)_m]^+$ clusters calculated at the UB3P86/def2-TZVPPD level of theory. Information included are harmonic frequencies, and cartesian coordinates of optimised structures. For the full set of anharmonic frequencies, please see the data availability statement and associated Oxford Research Archive link in the main article.

Table of Contents

1. Au_n^+ + CO_2 Reactivity 3
2. Harmonic frequencies of calculated $[Au_4CO_2]^+$ structures 4
3. Harmonic frequencies of calculated $[Au_5CO_2]^+$ structures 5
4. Harmonic frequencies of calculated $[Au_6CO_2]^+$ structures 7
5. Calculated structures of $[Au_4CO_2]^+$ 9
6. Calculated structures of $[Au_5CO_2]^+$ 10
7. Calculated structures of $[Au_6CO_2]^+$ 11
8. Calculated $[Au_4(CO_2)_2]^+$ structures 12
9. Calculated $[Au_4(CO_2)_3]^+$ structures 14

1. Au_n^+ + CO_2 Reactivity

As noted in the main manuscript, relative rate constants have been extracted from the mass spectral data through the intensity of the individual mass peaks, the data of which can be seen in Figure S1. The relative rate constants can be seen to decrease with increasing cluster size, an observation suggesting the binding of CO_2 relates to the overall charge of the metal cluster; a quantity that would indeed decrease with increasing cluster size. Furthermore, there does not appear to be any evidence of an odd-even cluster size dependence in the binding.

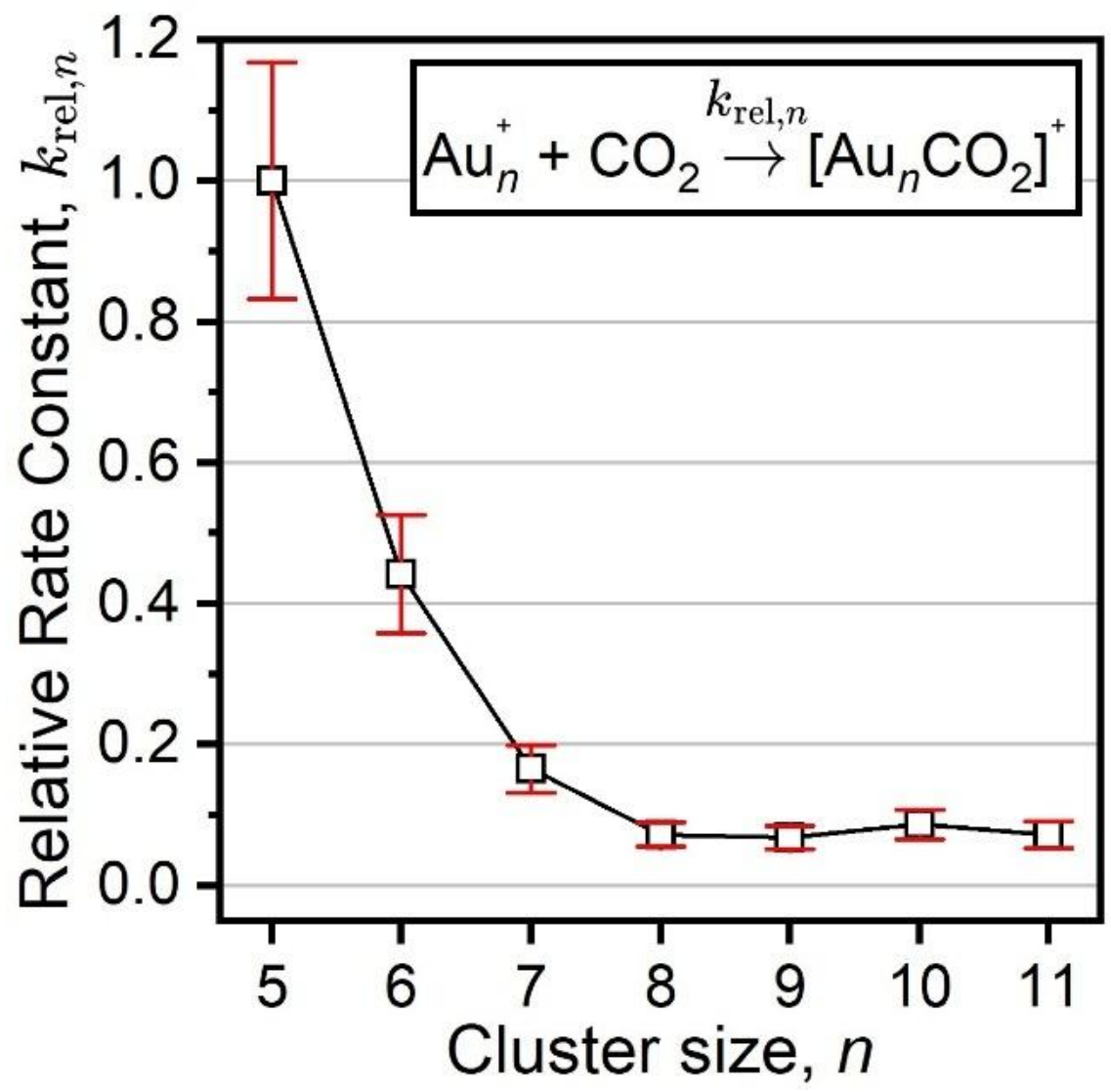

Figure S1: Relative rate constants associated with the reactivity of size-selected gold cluster cations with CO_2.

2. Harmonic frequencies of calculated $[Au_4CO_2]^+$ structures

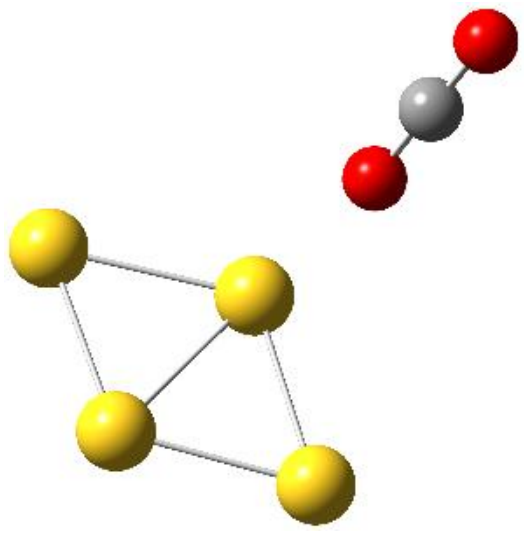

4A

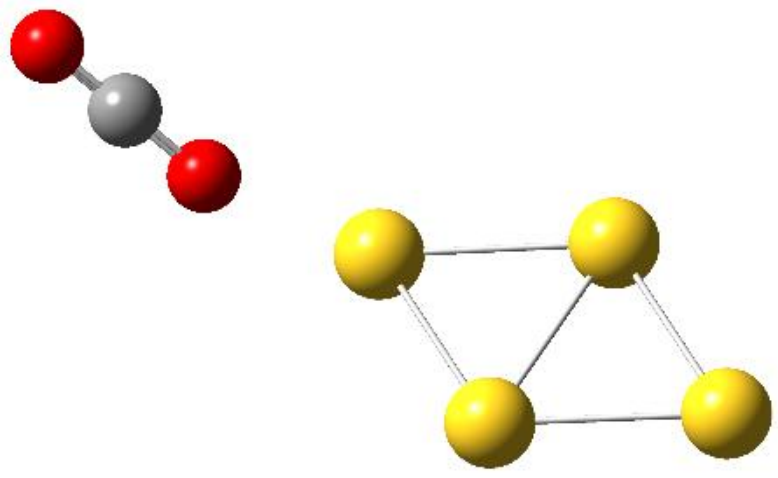

4B

Table S1: Calculated harmonic frequencies of the $[Au_4CO_2]^+$ structures in Figure 3a.

Structure	Mode	Frequency (cm^{-1})	Intensity (km/mol)
	ω_1	9.4	0.1
	ω_2	12.5	<0.1
	ω_3	26.4	0.1
	ω_4	30.1	0.1
	ω_5	67.6	0.3
	ω_6	76.4	0.5
	ω_7	84.8	0.9
4A	ω_8	103.8	0.0
	ω_9	145.8	0.1
	ω_{10}	147.5	4.4
	ω_{11}	183.9	14.3
	ω_{12}	656.3	22.3
	ω_{13}	666.2	24.1
	ω_{14}	1378.7	42.9
	ω_{15}	2441.8	1259.8
	ω_1	8.1	0.1
	ω_2	12.8	0.1
	ω_3	22.5	0.1
	ω_4	26.9	<0.1
	ω_5	64.8	0.7
	ω_6	81.5	1.1
	ω_7	83.2	<0.1
4B	ω_8	97.8	<0.1
	ω_9	137.1	10.6
	ω_{10}	154.9	4.3
	ω_{11}	177.9	21.4
	ω_{12}	658.6	26.4
	ω_{13}	666.6	24.0
	ω_{14}	1376.5	55.6
	ω_{15}	2438.5	1470.7

3. Harmonic frequencies of calculated $[Au_5CO_2]^+$ structures

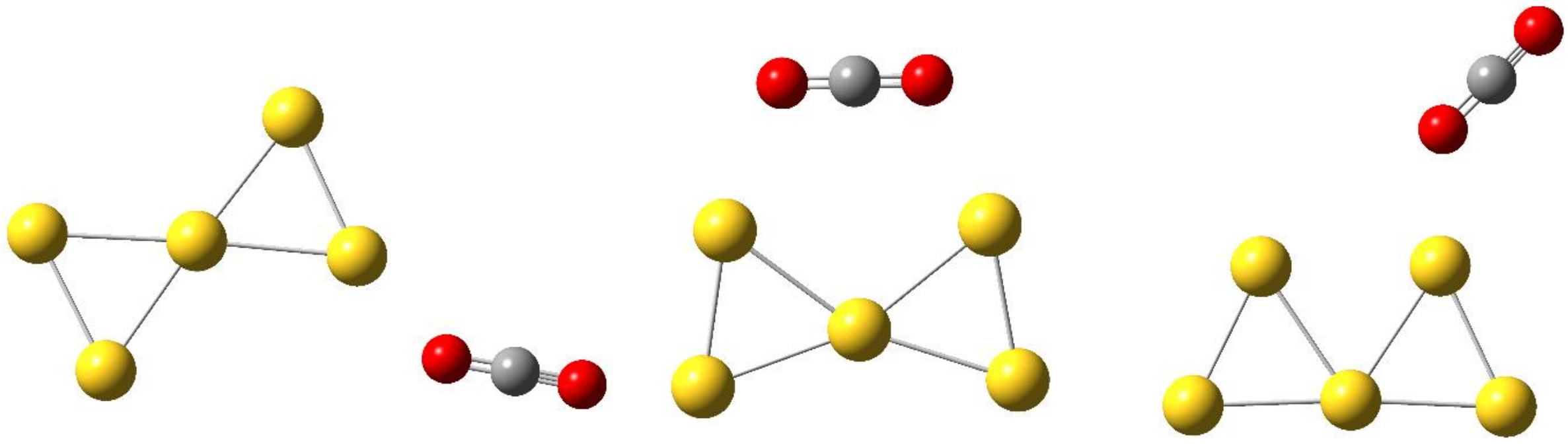

5A 5B 5C

Table S2: Calculated harmonic frequencies of the $[Au_5CO_2]^+$ structures in Figure 3b.

Structure	Mode	Frequency (cm^{-1})	Intensity (km/mol)
5A	ω_1	1.5	0.1
	ω_2	6.8	<0.1
	ω_3	15.6	0.3
	ω_4	24.6	<0.1
	ω_5	36.3	0.6
	ω_6	62.2	0.1
	ω_7	66.5	0.2
	ω_8	74.7	0.1
	ω_9	84.9	<0.1
	ω_{10}	112.1	0.4
	ω_{11}	133.3	0.1
	ω_{12}	157.4	4.4
	ω_{13}	181.3	9.0
	ω_{14}	204.1	8.3
	ω_{15}	654.7	27.4
	ω_{16}	667.6	22.1
	ω_{17}	1379.4	42.2
	ω_{18}	2442.9	1435.4
5B	ω_1	13.9	0.0
	ω_2	23.4	0.4
	ω_3	28.4	0.3
	ω_4	34.5	0.8
	ω_5	39.4	<0.1
	ω_6	68.9	0.0
	ω_7	74.9	<0.1
	ω_8	80.6	0.1
	ω_9	104.0	1.2
	ω_{10}	108.4	3.8
	ω_{11}	114.6	1.8
	ω_{12}	138.7	0.2
	ω_{13}	172.7	1.6
	ω_{14}	191.4	1.4
	ω_{15}	661.6	20.9
	ω_{16}	661.7	45.2

	ω_{17}	1370.7	4.5
	ω_{18}	2395.6	473.6
5C	ω_{1}	6.6	0.1
	ω_{2}	16.3	<0.1
	ω_{3}	20.3	<0.1
	ω_{4}	24.2	0.1
	ω_{5}	32.4	0.3
	ω_{6}	65.0	0.3
	ω_{7}	66.9	0.5
	ω_{8}	69.1	0.2
	ω_{9}	85.0	0.3
	ω_{10}	119.7	0.4
	ω_{11}	128.7	0.5
	ω_{12}	154.8	1.8
	ω_{13}	178.0	6.3
	ω_{14}	201.2	13.1
	ω_{15}	655.4	26.8
	ω_{16}	668.7	20.3
	ω_{17}	1375.9	46.0
	ω_{18}	2440.0	1393.5

4. Harmonic frequencies of calculated $[Au_6CO_2]^+$ structures

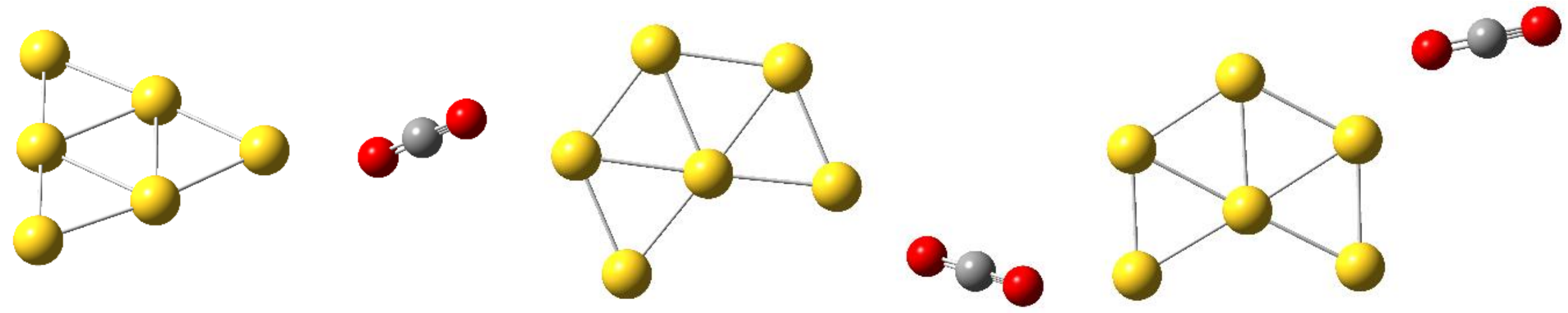

6A 6B 6C

Table S3: Calculated harmonic frequencies of the $[Au_6CO_2]^+$ structures in Figure 3c.

Structure	Mode	Frequency (cm^{-1})	Intensity (km/mol)
6A	ω_{1}	6.9	0.2
	ω_{2}	15.3	0.4
	ω_{3}	26.2	0.1
	ω_{4}	29.7	0.5
	ω_{5}	32.8	<0.1
	ω_{6}	35.8	0.1
	ω_{7}	38.9	0.3
	ω_{8}	68.1	0.3
	ω_{9}	69.2	0.2
	ω_{10}	82.3	0.2
	ω_{11}	83.8	0.4
	ω_{12}	114.7	0.2
	ω_{13}	133.0	1.1
	ω_{14}	141.7	0.3
	ω_{15}	160.5	27.1
	ω_{16}	180.2	3.4
	ω_{17}	192.7	6.8
	ω_{18}	658.9	24.9
	ω_{19}	667.5	24.7
	ω_{20}	1377.7	57.7
	ω_{21}	2438.8	1593.4
6B	ω_{1}	6.5	0.1
	ω_{2}	9.7	0.1
	ω_{3}	14.0	<0.1
	ω_{4}	22.7	0.1
	ω_{5}	36.2	0.4
	ω_{6}	40.4	0.1
	ω_{7}	50.6	0.1
	ω_{8}	63.6	0.1
	ω_{9}	67.0	0.5
	ω_{10}	74.1	0.1
	ω_{11}	99.5	0.1
	ω_{12}	104.9	0.2
	ω_{13}	128.5	1.1
	ω_{14}	140.0	2.2

	ω_{15}	143.4	2.8
	ω_{16}	166.2	22.2
	ω_{17}	190.3	6.8
	ω_{18}	658.8	24.4
	ω_{19}	667.9	23.5
	ω_{20}	1377.8	51.3
	ω_{21}	2438.7	1589.0
6C	ω_{1}	2.7	0.1
	ω_{2}	9.6	<0.1
	ω_{3}	14.9	<0.1
	ω_{4}	16.8	0.1
	ω_{5}	36.8	0.6
	ω_{6}	47.5	<0.1
	ω_{7}	53.2	0.5
	ω_{8}	56.6	<0.1
	ω_{9}	64.4	0.1
	ω_{10}	70.5	<0.1
	ω_{11}	95.8	0.6
	ω_{12}	108.1	0.3
	ω_{13}	126.3	2.4
	ω_{14}	130.2	8.7
	ω_{15}	146.7	4.9
	ω_{16}	153.4	8.0
	ω_{17}	188.1	1.3
	ω_{18}	661.6	26.7
	ω_{19}	670.1	20.9
	ω_{20}	1378.7	41.1
	ω_{21}	2438.2	1530.5

5. Calculated structures of $[Au_4CO_2]^+$

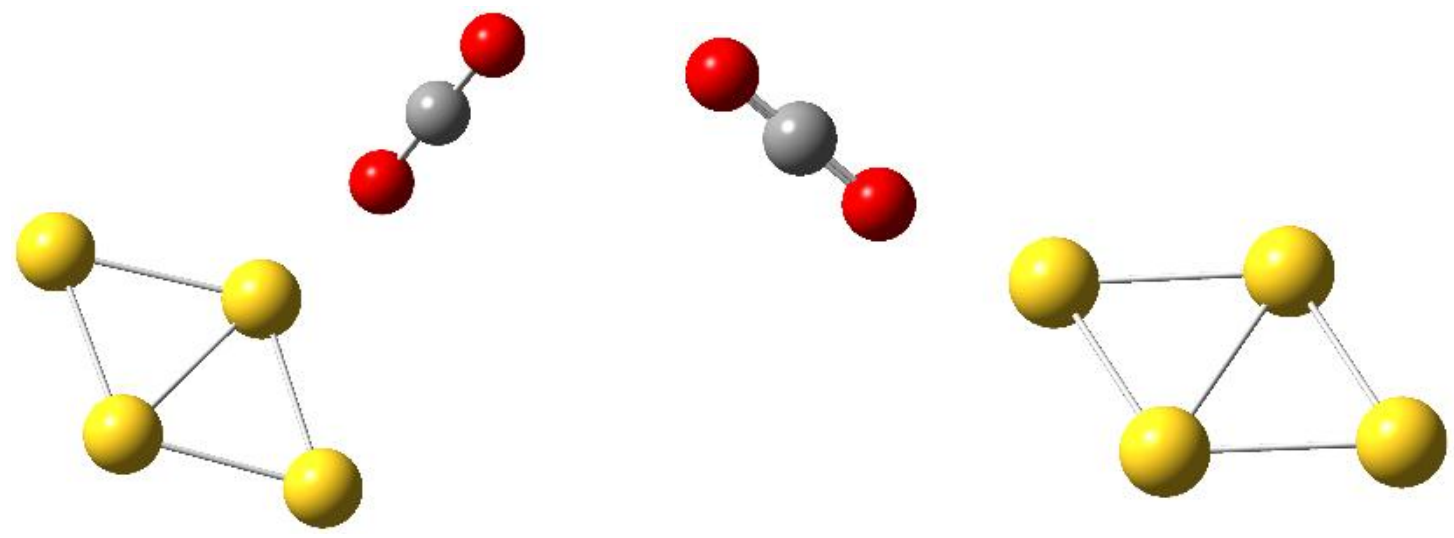

4A 4B

Table S4: Cartesian coordinates, in Å, of the $[Au_4CO_2]^+$ structures in Figure 3a.

Structure	Atom	X	Y	Z
4A	Au	-0.000542	1.047508	-0.141359
	Au	0.000875	-1.600739	0.129486
	Au	-2.311493	-0.330659	-0.019897
	Au	2.311887	-0.328270	-0.019852
	C	-0.002589	4.358323	0.195366
	O	-0.001322	3.347549	-0.396943
	O	-0.003908	5.353783	0.760197
4B	Au	0.360342	-1.329068	0.014279
	Au	0.359567	1.328829	0.014620
	Au	-1.936407	-0.000822	0.158715
	Au	2.674127	0.000667	-0.102400
	C	-5.239575	0.001418	-0.315442
	O	-4.264615	0.000397	0.334020
	O	-6.199797	0.002425	-0.938930

6. Calculated structures of $[Au_5CO_2]^+$

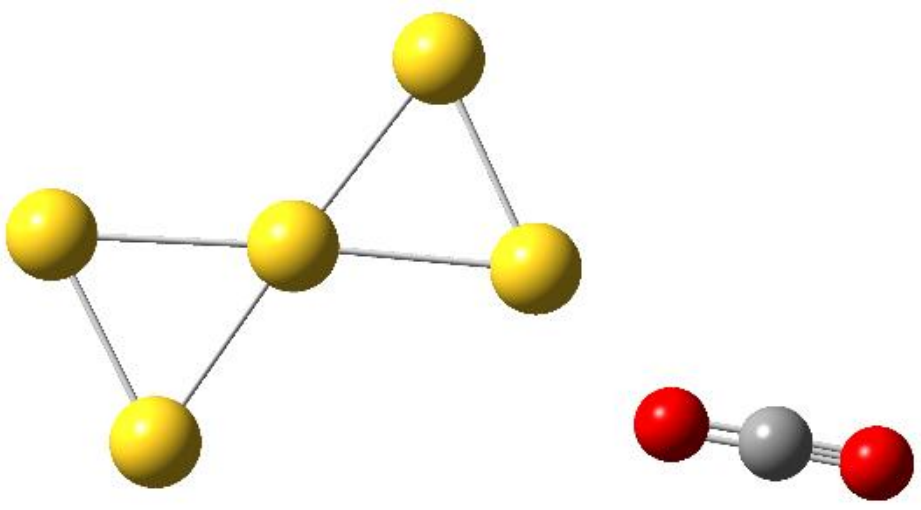

5A

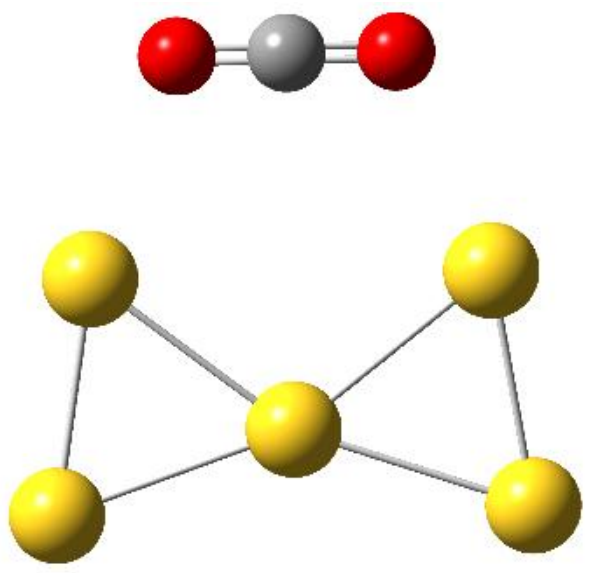

5B

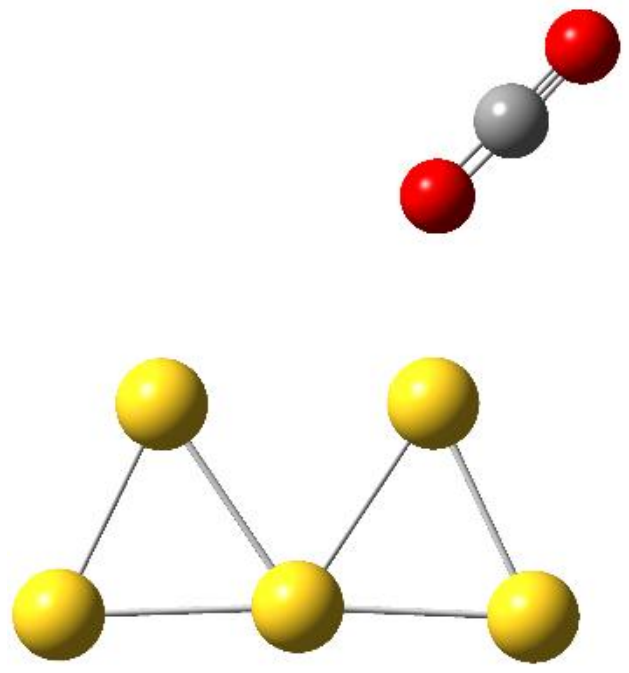

5C

Table S5: Cartesian coordinates, in Å, of the $[Au_5CO_2]^+$ structures in Figure 3b.

Structure	Atom	X	Y	Z
5A	Au	0.272010	-0.202933	0.001714
	Au	-2.343345	0.461168	0.006766
	Au	-1.656056	-2.007234	0.005593
	Au	2.128694	1.714625	0.013146
	Au	2.892325	-0.742042	-0.018399
	C	-4.656590	2.784628	-0.032317
	O	-5.732184	3.174960	-0.071759
	O	-3.549954	2.403675	0.008901
5B	Au	0.000032	-0.511983	0.000043
	Au	-2.120542	1.149015	-0.000049
	Au	-2.518979	-1.383501	-0.000018
	Au	2.120460	1.148976	-0.000148
	Au	2.518919	-1.383618	0.000078
	C	0.000393	3.529937	0.000375
	O	-1.158822	3.520436	0.000460
	O	1.159611	3.520585	0.000186
5C	Au	-0.460291	-1.001142	-0.000277
	Au	1.686991	0.658278	-0.000077
	Au	1.988441	-1.924053	0.000395
	Au	-1.180096	1.590763	0.001224
	Au	-3.008493	-0.254792	-0.000800
	C	3.508080	3.341252	-0.001718
	O	4.519083	3.876939	-0.002216
	O	2.462652	2.810225	-0.001094

7. Calculated structures of $[Au_6CO_2]^+$

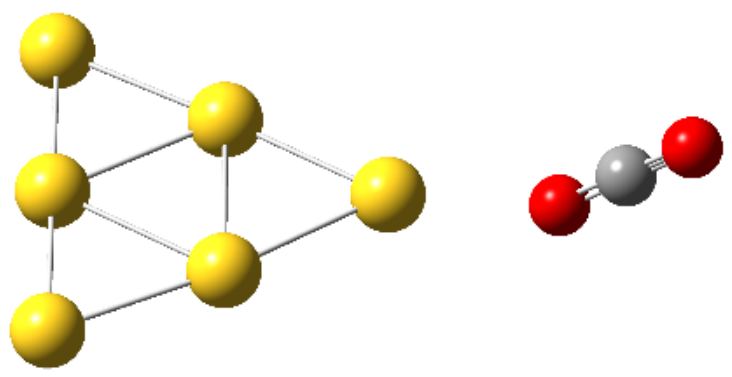

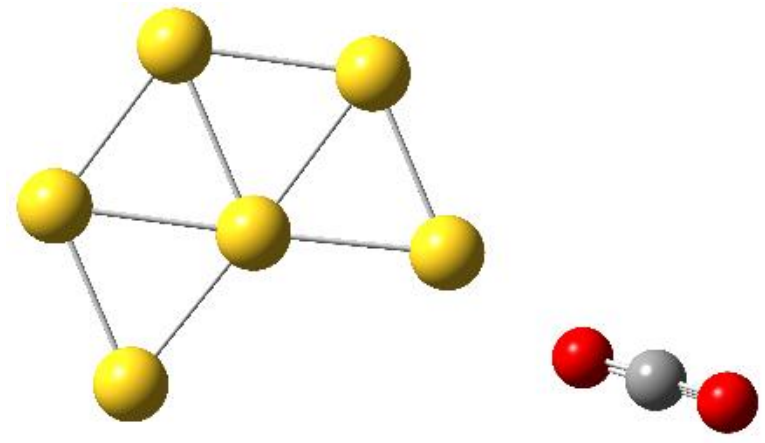

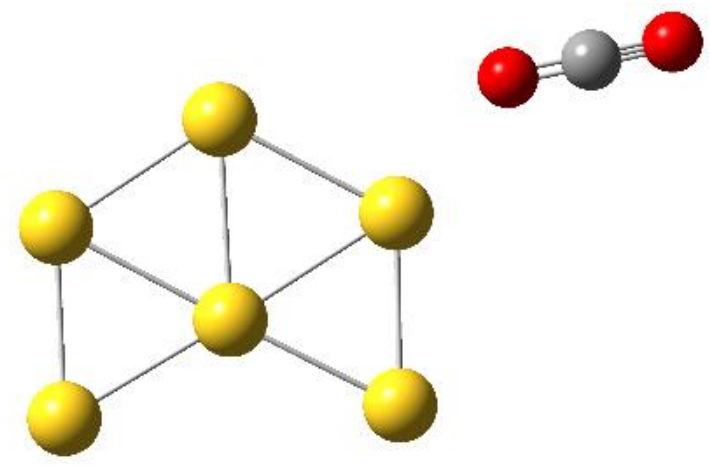

6A 6B 6C

Table S6: Cartesian coordinates, in Å, of the $[Au_6CO_2]^+$ structures in Figure 3c.

Structure	Atom	X	Y	Z
6A	Au	1.805274	0.081791	0.051530
	Au	-0.404524	-1.415613	-0.046547
	Au	1.969943	-2.526079	0.057501
	Au	-0.631991	1.396929	-0.052567
	Au	1.642405	2.692455	0.044447
	Au	-2.714703	-0.196578	-0.161880
	C	-5.988276	-0.117287	0.396115
	O	-5.037848	-0.191014	-0.283936
	O	-6.926676	-0.045955	1.048568
6B	Au	0.034842	0.347737	-0.032474
	Au	2.745470	0.123560	0.076866
	Au	1.589618	2.474586	-0.035986
	Au	1.231792	-2.119041	0.071134
	Au	-1.472140	-1.880737	-0.037580
	Au	-2.605222	0.479572	-0.142456
	C	-5.479763	2.060936	0.369509
	O	-6.424069	2.323814	0.962460
	O	-4.519173	1.801930	-0.247196
6C	Au	-0.358307	-0.544666	-0.000016
	Au	2.248201	0.184287	-0.000443
	Au	1.545277	-2.344851	-0.000591
	Au	0.288097	2.118635	-0.000877
	Au	-2.294658	1.363915	0.000296
	Au	-2.935655	-1.169540	0.000810
	C	5.419564	1.403898	0.002971
	O	4.254784	1.506830	-0.000393
	O	6.562607	1.313415	0.006260

8. Calculated $[Au_4(CO_2)_2]^+$ structures

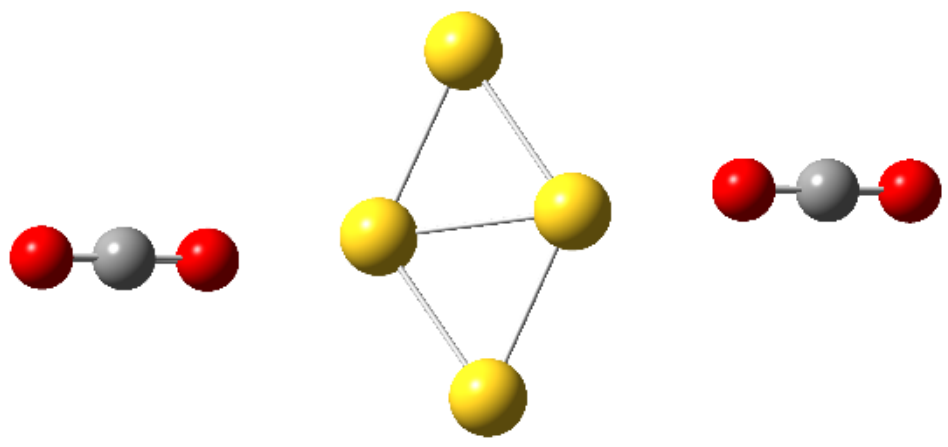

Table S7: Calculated harmonic frequencies of the $[Au_4(CO_2)_2]^+$ structure in Figure 5a.

Mode	Frequency (cm^{-1})	Intensity (km/mol)
ω_1	8.2	0.1
ω_2	8.7	0.1
ω_3	9.4	<0.1
ω_4	21.8	0.3
ω_5	29.9	<0.1
ω_6	36.3	0.1
ω_7	40.2	<0.1
ω_8	64.6	0.5
ω_9	70.0	0.0
ω_{10}	79.5	2.2
ω_{11}	82.4	<0.1
ω_{12}	109.7	0.0
ω_{13}	139.5	<0.1
ω_{14}	141.2	0.1
ω_{15}	161.2	38.8
ω_{16}	191.8	<0.1
ω_{17}	658.2	2.6
ω_{18}	658.3	41.0
ω_{19}	667.5	8.1
ω_{20}	667.6	40.8
ω_{21}	1379.1	80.0
ω_{22}	1379.8	0.2
ω_{23}	2437.7	2618.6
ω_{24}	2444.6	0.3

Table S8: Cartesian coordinates, in Å, of the $[Au_4(CO_2)_2]^+$ structure in Figure 5a.

Atom	X	Y	Z
Au	-1.308808	0.000800	0.172814
Au	1.309868	-0.003009	-0.170283
Au	0.002939	2.321920	-0.004287
Au	-0.003842	-2.323144	-0.004178
C	-4.643443	0.010104	-0.083174
O	-3.622046	0.010994	0.488468
O	-5.650069	0.009358	-0.629632
C	4.642825	0.002124	0.104483
O	3.624937	0.005207	-0.473371
O	5.646087	-0.000824	0.657155

9. Calculated $[Au_4(CO_2)_3]^+$ structures

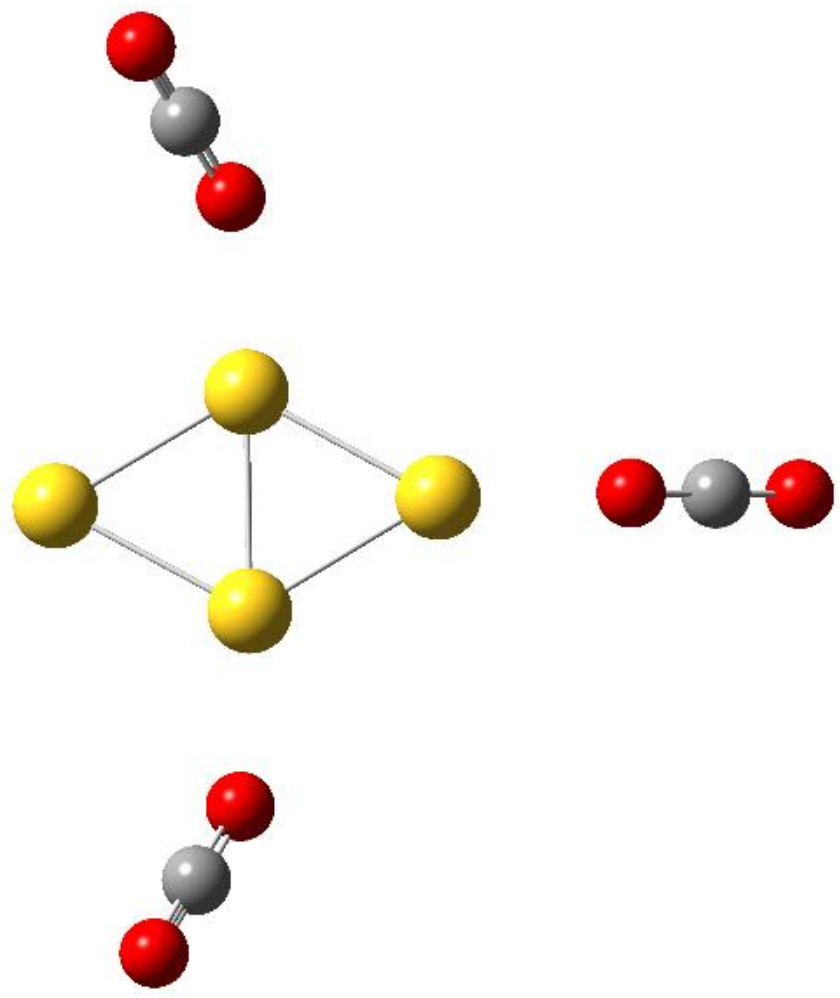

Table S9: Calculated harmonic frequencies of the $[Au_4(CO_2)_3]^+$ structure displayed in Figure 5b.

Mode	Frequency (cm^{-1})	Intensity (km/mol)
ω_1	6.0	<0.1
ω_2	6.2	0.1
ω_3	7.9	0.1
ω_4	9.3	<0.1
ω_5	17.7	0.3
ω_6	25.7	0.1
ω_7	26.2	<0.1
ω_8	33.4	0.2
ω_9	41.2	0.2
ω_{10}	47.1	<0.1
ω_{11}	61.1	0.4
ω_{12}	65.5	0.5
ω_{13}	70.8	<0.1
ω_{14}	76.8	2.8
ω_{15}	83.8	0.1
ω_{16}	106.6	0.0
ω_{17}	130.1	7.5
ω_{18}	139.1	1.3
ω_{19}	158.6	34.5
ω_{20}	164.5	25.8
ω_{21}	194.9	1.6
ω_{22}	659.0	0.1
ω_{23}	659.2	44.7
ω_{24}	660.5	24.7
ω_{25}	668.5	18.2
ω_{26}	668.5	13.6
ω_{27}	668.7	39.9
ω_{28}	1376.2	44.0
ω_{29}	1379.1	60.8
ω_{30}	1379.6	7.4

ω_{31}	2434.1	1904.6
ω_{32}	2436.6	2004.7
ω_{33}	2445.2	120.0

Table S10: Cartesian coordinates, in Å, of the $[Au_4(CO_2)_3]^+$ structure displayed in Figure 5b.

Atom	X	Y	Z
Au	0.245545	1.309717	-0.043672
Au	0.243781	-1.310317	-0.044878
Au	2.584385	-0.001677	-0.257978
Au	-2.065002	0.001071	0.149427
C	0.874129	4.540711	0.531874
O	0.398582	3.648059	-0.056029
O	1.334446	5.424214	1.098281
C	-5.353178	0.006143	-0.349319
O	-4.406171	0.003506	0.338007
O	-6.288132	0.008701	-1.012469
C	0.863481	-4.542498	0.532125
O	0.394415	-3.648884	-0.059520
O	1.317533	-5.426953	1.102093
